# The effectiveness of mindfulness-based interventions for children with autism and their parents: a systematic review and meta-analysis

**DOI:** 10.3389/fpsyg.2025.1526001

**Published:** 2025-06-27

**Authors:** Qiyan Peng, Yujie Dong, Jie Jin, Huan Ao, Chi Zhang, Yanfei Ma

**Affiliations:** ^1^Department of Nursing, Leshan Vocational and Technical College, Leshan, China; ^2^Department of Cardiology, West China Hospital, Sichuan University, Chengdu, China; ^3^Mianyang Central Hospital, School of Medicine, University of Electronic Science and Technology of China, Mianyang, Sichuan, China

**Keywords:** mindfulness-based therapies, autism spectrum disorder, systematic review, meta-analysis, parent–child intervention, intervention efficacy author country population year age

## Abstract

**Objective:**

This study aims to systematically evaluate the efficacy of mindfulness-based interventions (MBIs) for children diagnosed with autism spectrum disorder (ASD) and their parents.

**Methods:**

A comprehensive search of PubMed, Embase, Cochrane Library, Web of Science, PsycINFO and ERIC was conducted to identify randomized controlled trials (RCTs) published up to December 31, 2024, that assessed the effects of MBIs on children with ASD and their parents. Two independent reviewers screened studies, extracted relevant data, and assessed the quality of the included literature. A meta-analysis was performed using standardized mean differences (SMDs) and 95% confidence intervals (CIs).

**Results:**

A total of 12 RCTs involving 643 participants were included. The meta-analysis showed that MBIs significantly reduced parental stress [SMD = −0.69, 95% CI (−1.36, −0.02), *p* = 0.04], improved parental mindfulness awareness [SMD = 3.08, 95% CI (0.26, 5.90), *p* = 0.03], and alleviated anxiety, depression, and stress in parents [SMD = −0.57, 95% CI (−1.09, −0.06), *p* = 0.03]. Additionally, MBIs significantly improved social responsiveness in children with autism [SMD = −0.35, 95% CI (−0.66, −0.04), *p* = 0.03]. However, no statistically significant differences were observed between the MBI and control groups in reducing problematic behaviors in children [SMD = −0.45, 95% CI (−0.90, 0.00), *p* = 0.05], improving children’s emotional and behavioral difficulties [SMD = −0.23, 95% CI (−0.66, 0.19), *p* = 0.28], or enhancing parental psychological resilience [SMD = 0.85, 95% CI (−1.96, 3.66), *p* = 0.55].

**Conclusion:**

This meta-analysis demonstrates that MBIs significantly reduce parental stress, alleviate anxiety, depression, and stress, and enhance mindfulness awareness in parents of children with autism. Furthermore, MBIs were found to significantly improve social responsiveness in children with autism. However, their effects on children’s emotional and behavioral challenges and parental psychological resilience remain inconclusive.

**Systematic review registration:**

https://www.crd.york.ac.uk/PROSPERO/view/CRD42023424059.

## 1 Introduction

Autism spectrum disorder (ASD) is a complex neurodevelopmental condition characterized by deficits in social interaction, communication, and the presence of restricted and repetitive behaviors—features commonly described as the “Kanner triad” (Botelho et al., 2024; Lord et al., 2018). Symptoms typically emerge in early childhood. A recent global systematic review estimates that ASD affects approximately 1–2% of the world’s population, accounting for over 60 million individuals worldwide (Zeidan et al., 2022). Although ASD is more commonly diagnosed in males (male-to-female ratio of approximately 4:1), emerging research highlights potential diagnostic biases and gender-based phenotypic variability. Specifically, females with ASD often present with internalizing symptoms and employ stronger social camouflage strategies, contributing to underrecognition or delayed diagnoses (Lai et al., 2015; Schuck et al., 2019).

For many parents, receiving an ASD diagnosis for their child represents a profound psychological shift. Initial reactions commonly include denial, confusion, guilt, and grief, which are often part of a broader emotional process known as “diagnosis resolution” (Sher-Censor and Shahar-Lahav, 2022). These emotional responses can be shaped by cultural and contextual factors. In non-Western settings, such as Iran, parents report heightened stigma and emotional isolation following diagnosis (Sharifi et al., 2025). In atypical developmental contexts, like those involving hearing parents of oral deaf children, early relational disruptions may complicate parents’ understanding of their child’s emotional and mental states (Lecciso et al., 2013).

While a range of therapeutic approaches is available to address ASD’s core symptoms—including applied behavior analysis (ABA), cognitive behavioral therapy (CBT), speech and language therapy, and developmental social-pragmatic interventions—most demand intensive parental involvement (Vismara and Rogers, 2010; Wood et al., 2020). However, these interventions often overlook the emotional well-being of caregivers, a factor now recognized as critical to treatment adherence, family functioning, and child developmental outcomes.

Mindfulness-based interventions (MBIs), which integrate elements of mindfulness-based stress reduction (MBSR), acceptance and commitment therapy (ACT), and mindfulness-based cognitive therapy (MBCT), have gained attention as supportive strategies for families of children with ASD (Boyd et al., 2018; Schwartzman et al., 2022). For parents, MBIs offer tools to reduce stress, anxiety, and depressive symptoms while fostering emotion regulation and parenting self-efficacy. For children, particularly those with high emotional reactivity, modified MBIs may improve emotional awareness and social responsiveness (Kemeny et al., 2012; Ridderinkhof et al., 2018).

Although several systematic reviews have evaluated the effectiveness of MBIs, most have targeted either parents or children in isolation, without examining interrelated outcomes within family systems. For instance, Chua and Shorey (2022) and Suvarna et al. (2024) reported the efficacy of mindfulness- and ACT-based interventions in improving parental well-being but did not assess child-related outcomes. Other reviews (Borquist-Conlon et al., 2019; Li et al., 2023; Scherer et al., 2019) lacked recent randomized controlled trials (RCTs) or failed to adopt a dyadic focus. Therefore, the present study aims to update and extend existing evidence by including recent RCTs and concurrently evaluating the impact of MBIs on both children with autism and their parents. This dual focus offers a more integrated understanding of the familial impact of MBIs, thereby filling a critical gap in current literature.

## 2 Method

This systematic review was registered with the International Prospective Register of Systematic Reviews (PROSPERO) under registration number CRD42023424059.

### 2.1 Literature search methodology

A systematic search was conducted across six databases—PubMed, Web of Science, Embase, The Cochrane Library, PsycINFO and ERIC—for relevant studies published up to December 31, 2024. Gray literature, including dissertations, preprints, and trial registries such as ClinicalTrials.gov, was not included in this review. This may introduce a risk of publication bias, which is acknowledged as a limitation. To ensure comprehensive retrieval, broad search terms were used, focusing on the generic terms for “Autistic Disorder “and “Mindfulness.” The search strategy is detailed in Table 1 (using PubMed as an example).

**Table 1:** Search strategy on PubMed.

#1	“Mindfulness”[MeSH]
#2	Mindfulness Meditation[Title/Abstract] OR Meditation, Mindfulness[Title/Abstract] OR Mindfulness Meditations[Title/Abstract]
#3	#1 OR #2
#4	“Autistic Disorder “[MeSH]
#5	Disorder, Autistic[Title/Abstract] OR Disorders, Autistic[Title/Abstract] OR Autism[Title/Abstract] OR Autism, Early Infantile[Title/Abstract] OR Early Infantile Autism[Title/Abstract] OR Infantile Autism, Early[Title/Abstract] OR Autism, Infantile[Title/Abstract] OR Infantile Autism[Title/Abstract] OR Kanner’s Syndrome[Title/Abstract] OR Kanners Syndrome[Title/Abstract] OR Kanner Syndrome[Title/Abstract] “disorder autistic”[Title/Abstract] OR “disorders autistic”[Title/Abstract] OR “Autism”[Title/Abstract] OR “autism early infantile”[Title/Abstract] OR “early infantile autism”[Title/Abstract] OR “infantile autism early”[Title/Abstract] OR “autism infantile”[Title/Abstract] OR “infantile autism”[Title/Abstract] OR “kanner s syndrome”[Title/Abstract] OR “kanners syndrome”[Title/Abstract] OR “kanner syndrome”[Title/Abstract]
#6	#4 OR #5
#7	#3 AND #6

### 2.2 Inclusion and exclusion criteria

The inclusion and exclusion criteria for studies were based on the PRISMA statement and the “PICOS” framework, as follows: Population: Children diagnosed with autism and their parents; Intervention: Mindfulness-based therapy; Comparison: Control groups that did not receive any mindfulness intervention or placebo treatment; Outcomes: Psychological, emotional, and behavioral effects on children with autism and their parents; Study Design: Randomized controlled trials (RCTs).

Studies were excluded if they involved duplicate content or were non-randomized controlled trials (non-RCTs), including letters, animal studies, protocols, conference abstracts, and case reports. Additionally, studies were excluded if the intervention was not rooted in mindfulness-based practices.

### 2.3 Risk of bias and data extraction

The Cochrane Collaboration’s Risk of Bias tool was used to assess the risk of bias (Higgins et al., 2011). The following factors were considered to determine whether a study had a low, uncertain, or high risk of bias: random sequence generation and allocation concealment (selection bias); blinding of participants and personnel (performance bias); blinding of outcome assessment (detection bias); incomplete outcome data (attrition bias); selective reporting (reporting bias); and other potential biases. Two reviewers independently assessed and verified the quality of the included studies. In cases of disagreement, a third evaluation was conducted by the authors.

Two authors independently extracted the relevant data, which included the following: sample size, sex, and age of both experimental and control groups; first author; country of origin; year of publication; sample size; experimental intervention procedures and follow-up duration; control groups; and results.

### 2.4 Statistical analysis

Meta-analysis was performed using Review Manager version 5.4. Continuous variables were reported as mean difference (MD) or standardized mean difference (SMD), accompanied by 95% confidence intervals (CIs). Categorical data were expressed as relative risk (RR) with corresponding 95% CIs. A two-tailed test was applied to assess heterogeneity. A fixed-effects model was employed when *p* > 0.05 and *I*^2^ < 50%, indicating the absence of significant heterogeneity across studies. In cases of substantial heterogeneity (*p* < 0.05, *I*^2^ > 50%), a random-effects model was applied to estimate the cumulative effect size. Differences with *p* < 0.05 were considered statistically significant.

## 3 Results

### 3.1 Literature search results

**Figure 1:**
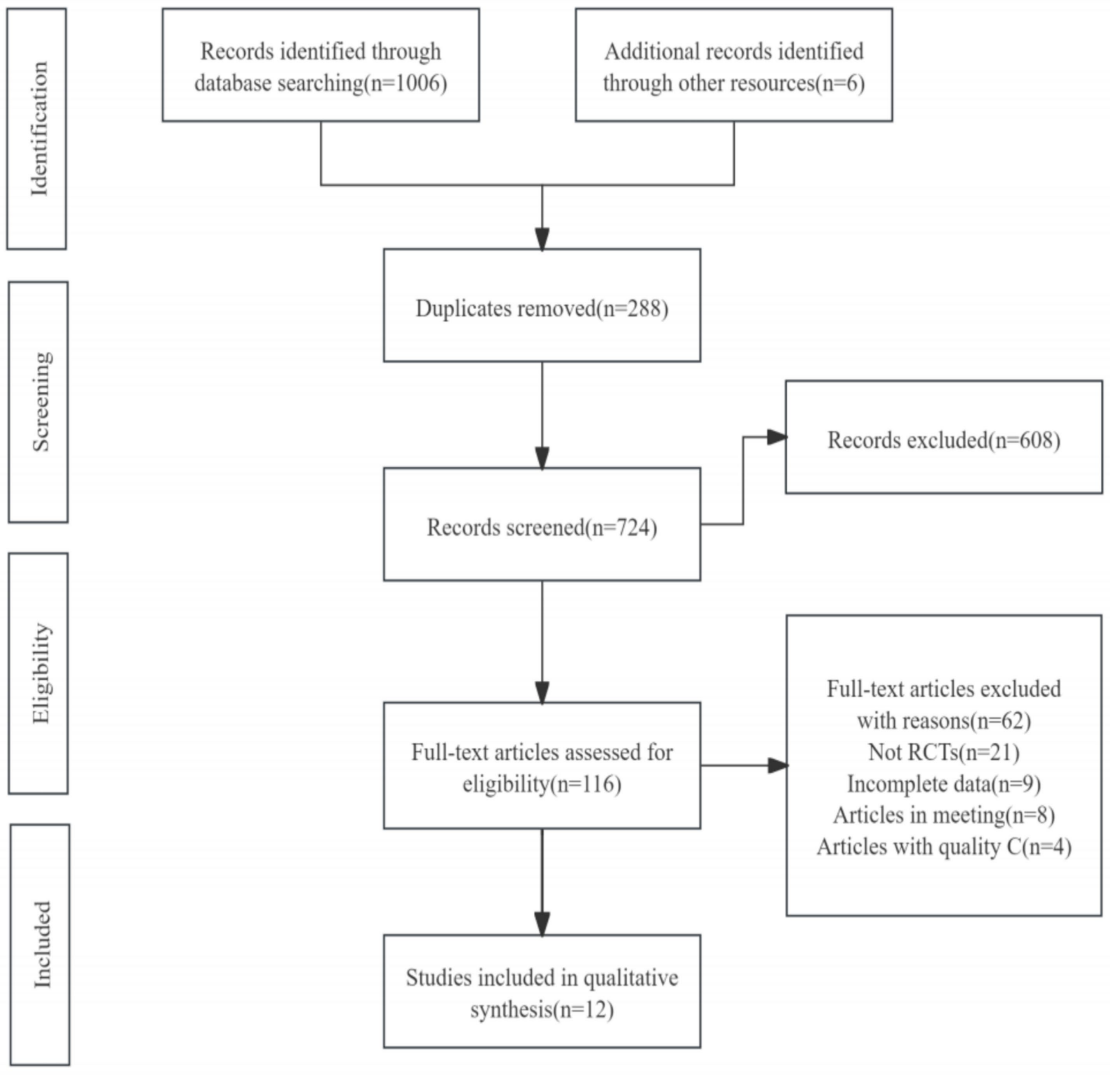
Flowchart of the literature selection process.

A total of 12 randomized controlled trials (RCTs) were included in this review, selected from 1,012 potentially eligible studies identified in the initial literature search. After removing duplicates, screening titles and abstracts, and reviewing full texts, studies that did not meet the inclusion criteria were excluded. The PRISMA flowchart illustrating the study selection process is presented in Figure 1. The detailed information of the included studies is provided in Table 2.

**Table 2:** General information of included studies.

Author	Country	Population	Year	Age (mean + SD)	Total/male/female	Intervention	Control	Outcome
Amy S. Weitlauf	USA	Parents of children with autism	2020	T: 33.27(6.24) C: 33.79(5.53)	T: 30/4/26 C: 31/4/27	P-ESDM + MBSR Length of Intervention: 6 months Freq: 12 times a week Duration: 1 h	P-ESDM	PSI-SF, CES-D, BAI, SLS, FFMQ
Neill Broderick	USA	Children with Autism	2022	T: 2.28(0.45) C: 2.28(0.45)	T:30/NA/NA C: 32/NA/NA	P-ESDM + MBSR Length of Intervention: 6 months Freq: 12 times a week Duration: 1 h	P-ESDM	ADOS-2, MSELVABS-II
Mohammad Saber Sotoodeh	Iran	Children with Autism	2017	T: 10.8(2.36) C: 10.5(1.87)	T: 15/11/4 C: 14/10/4	yoga training program Length of Intervention: 8 weeks Freq: 3 times a week Duration: 30 min	CON	ATEC
Sindhu Shanker	India	Children with Autism	2022	T: 9.77(2.63) C: 9.61(1.93)	T: 23/19/4 C: 20/16/4	Structured yoga Length of Intervention: 12 weeks Freq: 7 times a week Duration: 45 min	CON	SRS-2, ABC-2
Jessica M. Schwartzman	USA	Parents of children with autism	2022	T: 39.5(3.7) C: 42.5(5.4)	T: 17/4/13 C: 17/4/13	AMOR program Length of intervention: 8 weeks Freq: one time a week Duration: 90 min	DTG	CD-RISC-25, DASS-21, SRS-2, PSI-SF, AAQ-II, MAAS, LOF-R, ABC-2
Flavia Marino	Italy	Parents of children with autism	2021	T: 40.6(5.34) C: 42.0(5.71)	T: 20/NA/NA C: 20/NA/NA	ACT Length of Intervention: 6 months Freq: one time a week Duration: 90 min	PT	AAQ-II, HSQ-ASD, VLQ, MAAS, PSI-SF
Herman Hay Ming Lo	China	Parents of children with autism	2017	T: 39.31(NA) C: 38.40(NA)	T: 91/6/85 C: 89/5/84	Mindfulness practice Length of Intervention: 6 weeks Freq: one time a week Duration: 1.5 h	CON	PSI-SF, CESDS, ECBI, IMPS, KMSS
Leah R Ketcheson	USA	Parents of children with autism	2022	T: 37.54(8.89) C: 35.43(4.94)	T: 13/0/13\u00B0C: 14/1/13	mindfulness yoga program MYtime	CON	PSS, DASS
Ryan Yuk Fai Ho	China	Parents of children with autism	2021	T: 49.1(5.4) C: 44.1(5.5)	T: 19/6/13 C: 18/3/15	MYmind Length of Intervention: 9 weeks Freq: one time a week Duration: 90 min	CON	SRS, CBCL, BRIEF PSI-SF, PS, IM-P, WHO-5, RRS
A. Hemdi	UK	Children with autism and their parents	2017	Parents- T: 32.90(7.26) C: 34.43(6.65) Children-(month) T:63.18(13.68) C:58.73(14.07)	T: 32/0/32 C: 30/0/30	A Psychoeducation Intervention delivered via WhatsApp Length of Intervention: 8 weeks Freq: 5 times a week Duration: 60 min	CON	PSI-SF, HADS, SDQ, The Indian Scale for, Assessment of Autism, The Arabic Scale of Happiness
Adam D.	USA	Parents of children with autism	2019	T: 43.78 (4.63) C:47.22 (7.22)	T: 9/2/7 C: 9/3/6	ACT Intervention: 9 weeks Freq: one time a week Duration: 60 min	CON	AAQ-II, WBSI, ISS, CFQ-13, FMI, MAAS, PVQ-II, BDI-II
Pamela Clifford	Netherlands	Children with Autism	2022	T: 10.2 (1.58) C: 10.2 (1.56)	T: 26/20/6 C: 24/18/6	MBCT + DBT Length of Intervention: NA Freq: 9 times a week Duration: 60 min	CON	QSB, CBCL, TRF, NOSI-K, SCL-90, SRS, BARQ-C, PEDS QL

P-ESDM: Parent-implemented Early Start Denver Model; MBSR: Mindfulness-Based Stress Reduction; PSI-SF: Parenting Stress Index-Short Form; CES-D: Center for Epidemiologic Studies Depression Scale; BAI: Beck Anxiety Inventory; SLS: Satisfaction with Life Scale; FFMQ: Five Facet Mindfulness Questionnaire; ADOS-2: The Autism Diagnostic Observation Schedule—Second Edition; MSEL: The Mullen Scales of Early Learning; VABS-II: The Vineland Adaptive Behavior Scales—Second Edition; ATEC: The Autism Treatment Evaluation Checklist; SRS-2: Social Responsiveness Scale; Second Edition; ABC-2: Aberrant Behavior Checklist, Second Edition; AMOR: Acceptance, Mindfulness, Optimism, Resilience; DTG: Delayed Treatment Group; CD-RISC-25: Connor-Davidson Resilience Scale—25-item version; DASS-21: Depression Anxiety Stress Scales, 21-item version; AAQ-II: Acceptance and Action Questionnaire, Second Edition; MAAS: Mindful Attention Awareness Scale; LOF-R: Life Orientation Test, Revised; ACT: Acceptance and Commitment Therapy; PT: Parent Training; HSQ-ASD: Home Situation Questionnaire; VLQ: Valued Living Questionnaire; CESDS: The Center for Epidemiologic Studies Depression Scale; ECBI: The Eyberg Child Behavior Inventory; IMPS: The Interpersonal Mindfulness in Parenting Scale; KMSS: The Kansas Marital Satisfaction Scale; MYtime: Mindfulness Yoga Program; PSS: The Perceived Stress Scale; MYmind: Mindfulness Program; CBCL: The Child Behavior Checklist; BRIEF: The Behavior Rating Inventory of Executive Function; PS: Parenting Scale; IM-P: The Interpersonal Mindfulness in Parenting; WHO-5: WHO Well-being Index—5-item version; RRS: The Rumination Response Scale; HADS: Hospital Anxiety and Depression Scale; SDQ: Strengths and Difficulties Questionnaire; WBSI: The White Bear Suppression Inventory; ISS: The Internalized Shame Scale; CFQ-13: The Cognitive Fusion Questionnaire—13 Items; FMI: The Freiburg Mindfulness Inventory; PVQ-II: The Personal Values Questionnaire—Second Edition; BDI-II: The Beck Depression Inventory—Second Edition; MBCT: Mindfulness-Based Cognitive Therapy; DBT: Dialectical Behavior Therapy; QSB: The Questionnaire of Social Behavior; TRF: Teacher Version of the CBCL; NOSI-K: The Short, Dutch Version of the PSI, Parenting Stress Index; SCL-90: The Symptom Checklist-90; BARQ-C: The Behavioral Anger Response Questionnaire for Children; PEDS QL: Pediatric Quality of Life.

### 3.2 Risk for bias

**Figure 2:**
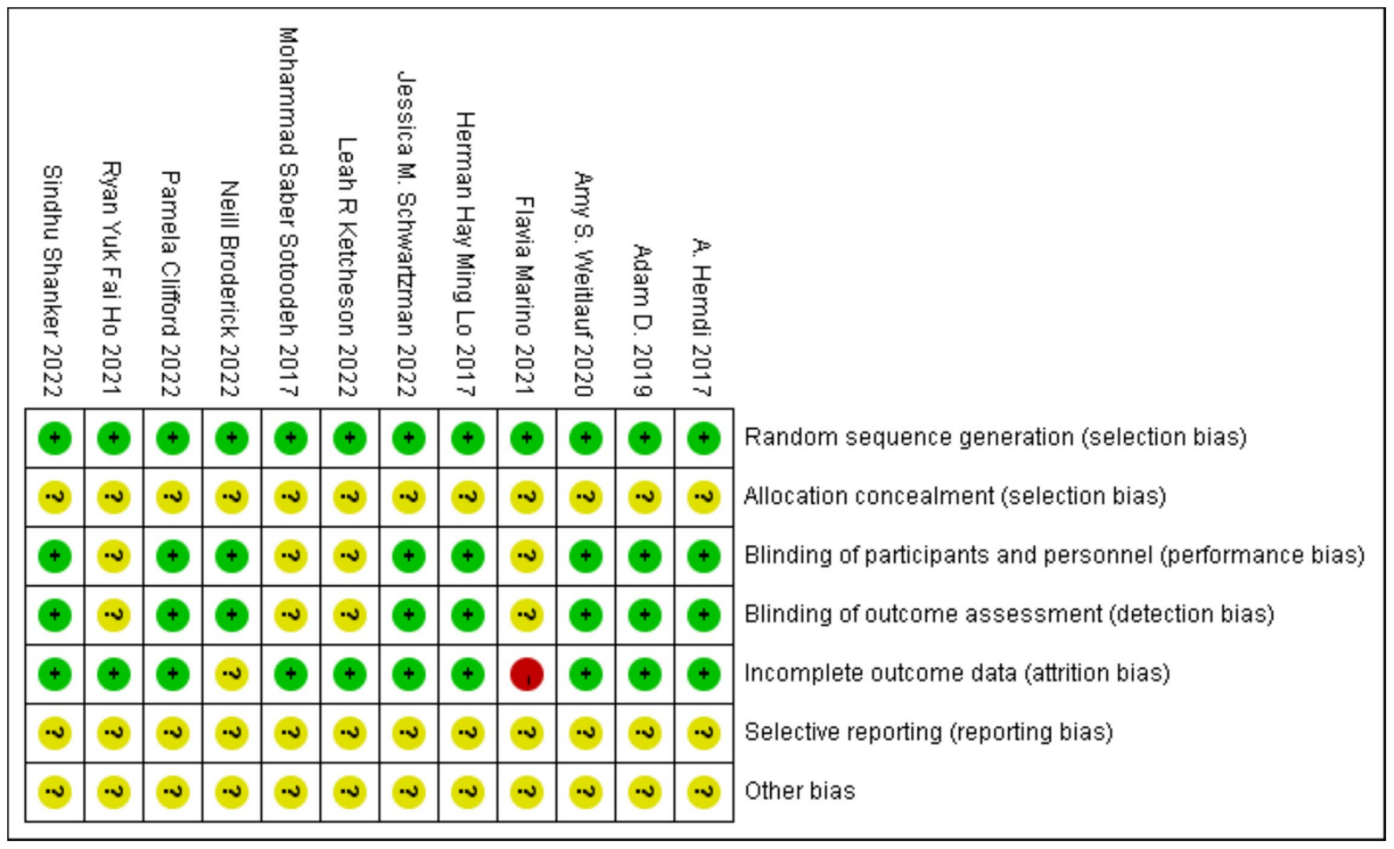
Risk of bias summary.

The included randomized controlled trials (RCTs) showed no significant baseline differences between the experimental and control groups. None of the 12 studies reported allocation concealment in their descriptions of the random sequence generation process. Eight studies documented the blinding of both participants and outcome assessors. One study reported a missing population and provided detailed handling of data for the disenrolled participants, whereas another study also had a missing population but did not describe how the data were managed. None of the 12 studies explicitly acknowledged any other sources of bias. Overall, the quality of the included studies was relatively reliable, although certain limitations were present. Specific risk assessment results are shown in Figure 2.

### 3.3 Meta analysis

#### 3.3.1 Effect of MBIs on parental stress in children with autism

**Figure 3:**
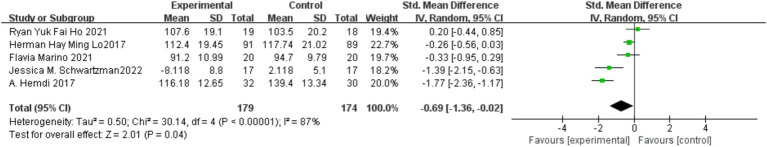
Impact of mindfulness-based interventions on parental stress in autism.

Five studies (Hemdi and Daley, 2017; Ho et al., 2021; Marino et al., 2021; Schwartzman et al., 2022) reported on the impact of MBIs on parental stress in children diagnosed with autism, with a total of 179 participants in the experimental group and 174 in the control group. Considerable heterogeneity was observed among the studies (I^2^ = 87%, *p* < 0.00001), so a random-effects model was applied. Since the measurement methods differed across studies, standardized mean differences (SMD) were used for the meta-analysis. The results indicated that the MBI intervention significantly reduced parental stress in parents of children with autism [SMD = −0.69, 95% CI (−1.36, −0.02), *p* = 0.04]. The details are presented in Figure 3.

#### 3.3.2 Effect of MBIs on parental psychological resilience in children with autism

**Figure 4:**
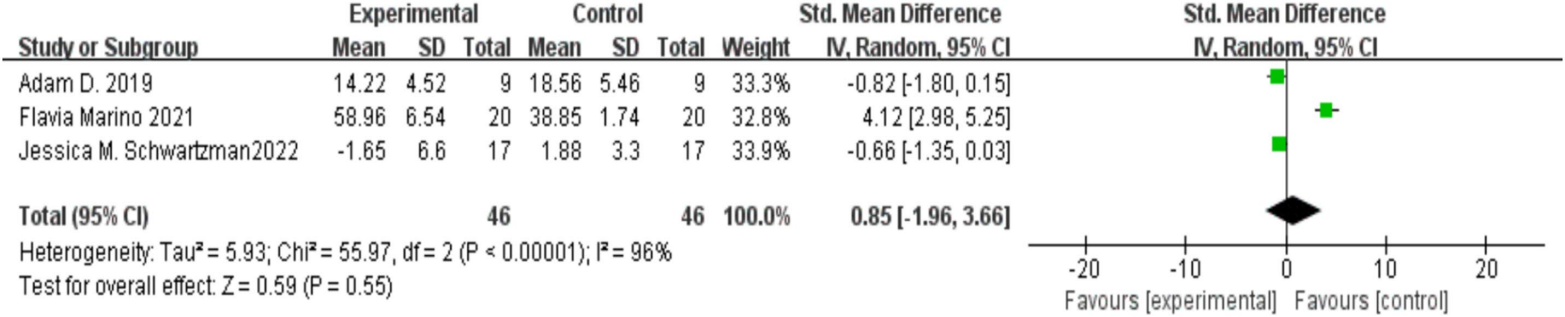
Effect of mindfulness-based interventions on parental psychological resilience in autism.

Three studies (Hahs et al., 2018; Marino et al., 2021; Schwartzman et al., 2022) reported on the impact of MBIs on the psychological resilience of parents of children with autism, with a total of 46 participants in both the experimental and control groups. Considerable heterogeneity was observed among the studies (*I*^2^ = 96%, *p* < 0.00001), so a random-effects model was applied. Since the measurement methods differed across studies, standardized mean differences (SMD) were used for the meta-analysis. The results indicated that the MBI intervention had no significant effect on the psychological resilience of parents of children with autism [SMD = 0.85, 95% CI (−1.96, 3.66), *p* = 0.55]. The details are presented in Figure 4.

#### 3.3.3 Impact of MBIs on the daily consciousness state of parents of children with autism

**Figure 5:**
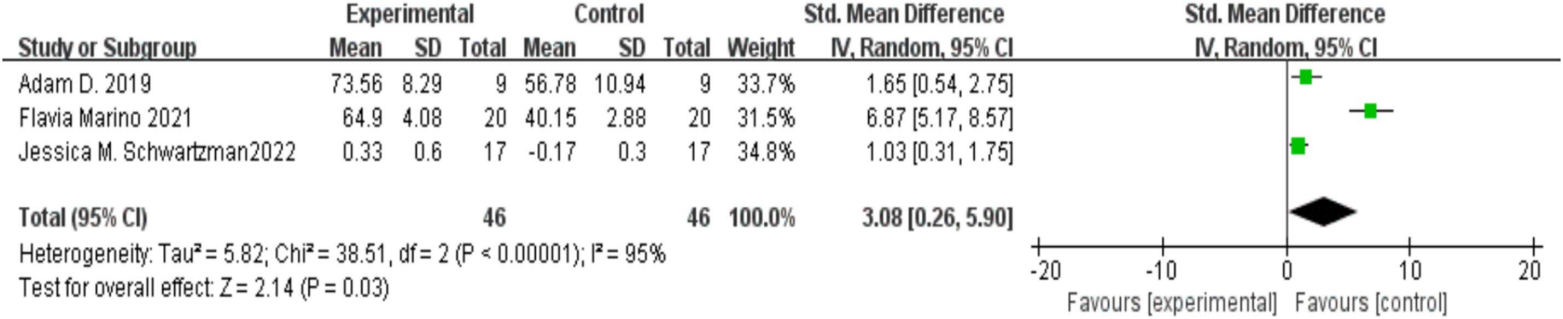
Impact of mindfulness-based interventions on parents’ daily consciousness in autism.

Three studies (Hahs et al., 2018; Marino et al., 2021; Schwartzman et al., 2022) investigated the effect of MBIs on the daily state of consciousness in parents of children with autism, with 46 participants in both the experimental and control groups. Significant heterogeneity was found across the studies (*I*^2^ = 95%, *p* < 0.00001), prompting the use of a random-effects model. The meta-analysis revealed that parents in the MBI group had significantly higher levels of consciousness than those in the control group [SMD = 3.08, 95% CI (0.26, 5.90), *p* = 0.03]. The results are illustrated in Figure 5.

#### 3.3.4 Effects of MBIs on anxiety, depression, and stress in parents of children with autism

**Figure 6:**
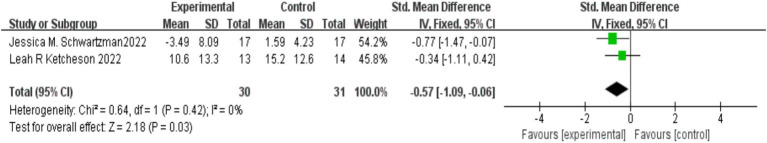
Effect of mindfulness-based interventions on anxiety, depression, and stress in parents of children with autism.

Two studies (Ketcheson et al., 2022; Schwartzman et al., 2022) reported the effects of MBIs on anxiety, depression, and stress in parents of children with autism, involving 30 participants in the experimental group and 31 in the control group. No significant heterogeneity was observed between the studies (I^2^ = 0%, *p* = 0.42), so a fixed-effects model was applied. The results showed that the MBI group exhibited significantly lower levels of anxiety, depression, and stress compared to the control group [SMD = −0.57, 95% CI (−1.09, −0.06), *p* = 0.03]. The details are shown in Figure 6.

#### 3.3.5 Effects of MBIs on the social responsiveness of children with autism

**Figure 7:**
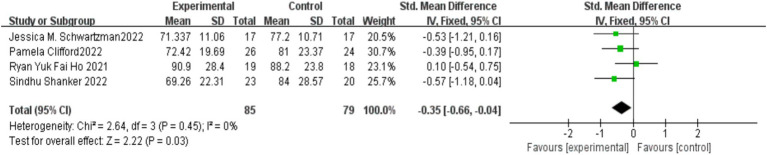
Effect of mindfulness-based interventions on social responsiveness in children with autism.

Four studies (Clifford et al., 2022; Ho et al., 2021; Schwartzman et al., 2022; Shanker and Pradhan, 2023) examined the impact of MBIs on the social responsiveness of children with autism, with 85 participants in the experimental group and 79 in the control group. No significant heterogeneity was observed between the studies (*I*^2^ = 0%, *p* = 0.45), and a fixed-effects model was applied. The meta-analysis revealed that MBIs significantly enhanced the social responsiveness of children with autism, with the difference between the groups being statistically significant [SMD = −0.35, 95% CI (−0.66, −0.04), *p* = 0.03]. The results are presented in Figure 7.

#### 3.3.6 Effects of MBIs on problematic behaviors in children with autism

**Figure 8:**
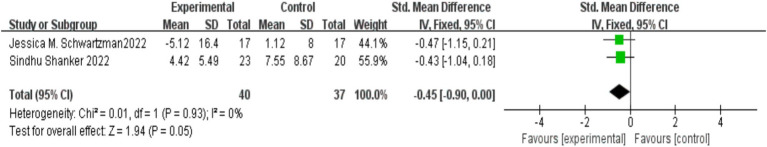
Effect of MBIs on externalized problem behaviors in children with autism.

Two studies (Schwartzman et al., 2022; Shanker and Pradhan, 2023; Ho et al., 2021) examined the effects of MBIs on problematic behaviors in children with autism, with 40 participants in the experimental group and 37 in the control group. No significant heterogeneity was found between the studies (*I*^2^ = 0%, *p* = 0.93), and a fixed-effects model was applied for the meta-analysis. The results indicated that there was no statistically significant difference between the mindfulness-based psychological intervention group and the control group [SMD = −0.45, 95% CI (−0.90, 0.00), *p* = 0.05]. The findings are illustrated in Figure 8.

#### 3.3.7 Effects of MBIs on emotional and behavioral problems in children with autism

**Figure 9:**
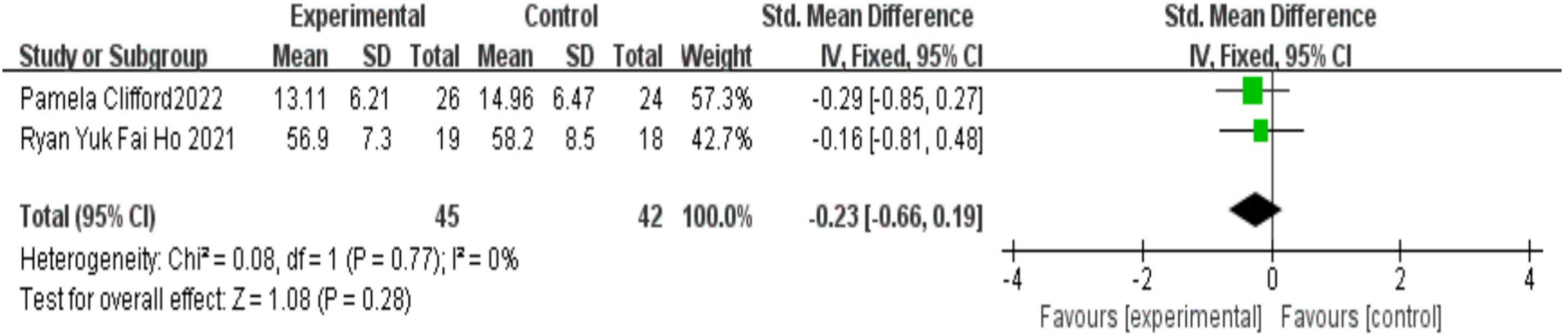
Effect of MBIs on broader emotional and behavioral difficulties in children with autism.

Two studies (Clifford et al., 2022; Ho et al., 2021) assessed the effects of MBIs on emotional and behavioral issues in children with autism, with 45 participants in the experimental group and 42 in the control group. No significant heterogeneity was observed between the studies (*I*^2^ = 0%, *p* = 0.77). Meta-analysis using a fixed-effects model indicated that the difference in the improvement of emotional and behavioral problems between the MBI group and the control group was not statistically significant [SMD = −0.23, 95% CI (−0.66, 0.19), *p* = 0.28]. The detailed results are presented in Figure 9.

### 3.4 Publication bias and sensitivity analysis

Visual inspection of funnel plots suggested approximate symmetry indicating low risk of publication bias for primary outcomes. However, we acknowledge that funnel plot interpretation is limited when few studies are included (<10 per outcome) and heterogeneity is high. Sensitivity analyses using the leave-one-out method confirmed that summary estimates for significant outcomes (e.g., parental stress, social responsiveness) remained robust and directionally consistent (all *p* < 0.05). Nevertheless, substantial heterogeneity persisted (*I*^2^ > 85%) even after sequentially removing individual studies, particularly for parental resilience and stress outcomes.

We attempted subgroup analyses to explore sources of heterogeneity (e.g., intervention duration, ASD severity), but insufficient reporting in original studies precluded meaningful stratification—only 4/12 studies specified ASD severity levels, and intervention durations varied widely (6 weeks to 6 months).

## 4 Discussion

This meta-analysis synthesized the results of 12 RCTs involving 643 children with autism and their parents, evaluating the impact of MBIs. The findings suggest that MBIs are a promising therapeutic tool, with significant positive effects on both the emotional well-being of parents and the behavioral outcomes of children with autism. Specifically, MBIs significantly reduced parental stress and anxiety, improved emotional regulation, and enhanced the social responsiveness of children. These results align with previous studies, such as those by Singh et al. (2019), which also highlighted the positive effects of mindfulness practices on stress reduction and emotional well-being in parents. By incorporating mindfulness practices into daily routines, parents reported enhanced emotional regulation, which likely facilitated more effective caregiving and improved family dynamics. This result aligns with Weitlauf et al. (2020), who demonstrated that MBIs help reduce parental distress and foster resilience in the face of caregiving challenges.

Mindfulness-based interventions offer parents effective strategies for managing stress and regulating emotions. Engaging in mindfulness exercises enables parents to become more aware of their emotional responses and stress levels, fostering better coping mechanisms. This aligns with findings by Aydin (2023), who demonstrated that mindfulness practices help parents not only acknowledge but also accept their emotions, thus preventing negative emotions like anxiety and despair from overwhelming them. Moreover, mindfulness exercises promote self-reflection, enabling parents to apply positive emotion regulation strategies and reduce stress while interacting with their children with autism (Mo et al., 2024).

One key observation in this study is that MBIs significantly enhance parents’ daily awareness. The increased mindfulness allows parents to better focus on their present emotional state and interactions with their children, improving their ability to understand and respond to their children’s needs. This heightened consciousness, in turn, leads to more effective communication and improved emotional bonding (Chua and Shorey, 2022). Thus, mindfulness interventions do not only reduce stress but also improve the overall quality of parent–child interactions.

Regarding children, the results indicate that MBIs can lead to improvements in emotional regulation and social behavior. The differential effects observed—where MBIs improve social responsiveness but not broader emotional or behavioral issues—may be attributed to the nature of mindfulness training, which emphasizes present-moment awareness and interpersonal attunement, directly benefiting social skills but less effective in altering entrenched emotional or behavioral patterns. This aligns with the dual-process theory of ASD (Ridderinkhof et al., 2021), where top-down cognitive control (targeted by MBIs) improves social attention, whereas bottom-up limbic dysregulation requires more intensive intervention. Children who participated in the mindfulness interventions showed notable improvements in social responsiveness and reduced stereotypical behaviors, which is consistent with findings from previous studies examining mindfulness interventions for children with ASD (Ridderinkhof et al., 2021). The improvement in social engagement is particularly important given that children with ASD often struggle with social communication and emotional regulation. The results suggest that mindfulness, particularly when adapted for children, may provide a useful tool in addressing these core deficits of ASD.

However, the effectiveness of MBIs varied depending on several factors, including the age of the child and the severity of their symptoms. Younger children, due to developmental limitations in attention span and emotional regulation, may have more difficulty engaging in traditional mindfulness practices. Older children, on the other hand, might benefit more significantly from the intervention due to their greater ability to focus and regulate their emotions (Borquist-Conlon et al., 2019; Tao et al., 2021). This age-dependent variability in intervention outcomes suggests that modifications to mindfulness practices may be necessary to optimize their effectiveness for different age groups.

Furthermore, it is important to note that the duration of the intervention and the specific mindfulness techniques used could have influenced the observed outcomes. Several studies have suggested that longer-duration mindfulness programs may yield better results, especially in improving behavioral outcomes in children (Xie et al., 2022). Our findings indicate that children who underwent longer mindfulness interventions experienced more substantial improvements in social and emotional domains. Therefore, further research is needed to determine the optimal duration and structure of mindfulness interventions for children with ASD.

While the results are promising, several limitations should be considered. It is noteworthy that all included studies were conducted in Western or Asian countries, raising concerns about cultural generalizability. Cultural norms fundamentally shape parenting practices and emotional expression—key mechanisms through which MBIs operate (Kirmayer, 2015)—potentially moderating intervention efficacy. Future research must therefore prioritize underrepresented regions (e.g., Africa, Latin America) and examine cultural adaptations of MBIs. First, the majority of studies included in this review were of moderate methodological quality, and many lacked long-term follow-up data. As a result, it is unclear whether the observed improvements in children’s behavior and parents’ well-being are sustained over time. Additionally, the lack of consistency in intervention delivery across studies—such as variations in the number of sessions, the intensity of the intervention, and the experience of the facilitators—makes it difficult to draw definitive conclusions about the most effective approach. Future research should aim to standardize these intervention parameters to better understand their impact. Third, the variation in intervention duration and intensity (ranging from 6-week to 6-month programs) may explain inconsistencies in findings. Unfortunately, due to reporting limitations, we could not formally assess this through subgroup analysis. Future studies should systematically examine dosage-response relationships.

In conclusion, the findings of this systematic review and meta-analysis support the use of mindfulness-based interventions as a promising approach for improving the emotional well-being of parents and the behavioral outcomes of children with ASD. However, further research is needed to examine the long-term effects of these interventions, as well as to explore how variables such as age, intervention duration, and severity of ASD symptoms influence the outcomes of mindfulness interventions. By optimizing the delivery of MBIs and understanding their underlying mechanisms, it is possible to enhance the therapeutic potential of these interventions in both children with ASD and their families.

This systematic review highlights the potential benefits of MBIs for children with autism spectrum disorder (ASD) and their parents. While these findings are promising, two critical considerations warrant emphasis: First, methodological limitations in the extant literature—particularly the frequent absence of allocation concealment and blinding in included RCTs—may contribute to overestimation of intervention effects, necessitating cautious interpretation of current evidence. Second, the broader field of MBIs for ASD remains developmental; though recent studies have expanded applications to high-functioning subgroups and diverse cultural contexts (Boyd et al., 2022; Zeidan et al., 2022), substantial knowledge gaps persist regarding intervention refinement, cultural adaptation, and long-term efficacy. These limitations collectively underscore the imperative for more rigorously designed trials that address methodological weaknesses while advancing context-specific implementation frameworks.

## 5 Conclusion

This systematic review and meta-analysis provides robust evidence that MBIs are effective in alleviating parental stress, anxiety, and depressive symptoms, while significantly enhancing mindfulness awareness among parents of children with autism. Furthermore, MBIs have demonstrated a moderate but meaningful impact on improving social responsiveness in children with ASD, suggesting their potential to address core social–emotional challenges. Clinically, MBIs may serve as an effective adjunctive therapy for reducing caregiver burden in ASD families, potentially enhancing adherence to developmental interventions and improving overall family functioning.

However, the analysis also revealed non-significant effects on reducing children’s problematic behaviors, improving emotional and behavioral difficulties, and enhancing parental psychological resilience. The parental resilience outcome exemplifies how meta-analytical imprecision (CI width >2 SDs) can mask clinically relevant signals. While statistically inconclusive, the point estimate (SMD = 0.85) aligns with qualitative reports of improved coping. Future trials require ≥80% power to detect resilience changes—our calculations suggest 150 participants/arm for SMD = 0.4. These inconclusive findings highlight the variability in outcomes, which may be influenced by factors such as child age, symptom severity, intervention type, and duration. Importantly, most included studies were of moderate methodological quality and lacked long-term follow-up, limiting the generalizability and sustainability of observed benefits. Moreover, the heterogeneity in delivery formats and assessment tools presents challenges in determining standardized best practices.

Future research should prioritize high-quality, longitudinal RCTs that incorporate dyadic designs, consider cultural and developmental adaptations, and explore synergistic effects with other evidence-based interventions. By optimizing the structure and personalization of MBIs, researchers and clinicians may better support the mental health of both children with ASD and their caregivers, contributing to more holistic and family-centered care strategies.

## Data Availability

The original contributions presented in the study are included in the article/supplementary material, further inquiries can be directed to the corresponding author.
